# Effects of Hypoxia on Acoustic Activity of Two Stored-Product Pests, Adult Emergence, and Grain Quality

**DOI:** 10.1093/jee/toz110

**Published:** 2019-05-14

**Authors:** Anastasia W Njoroge, Richard W Mankin, Bradley Smith, Dieudonne Baributsa

**Affiliations:** 1 Department of Entomology, Purdue University, West Lafayette, IN; 2 United States Department of Agriculture, Agricultural Research Service Center for Medical, Agricultural and Veterinary Entomology, Gainesville, FL

**Keywords:** modified atmosphere, hermetic storage, insect pest, cowpea, wheat

## Abstract

Modified atmospheres such as hermetic storage are widely used for the control of stored grain insect pests. To improve their effectiveness, there is need to better understand insect responses to low-oxygen environments. Adult *Callosobruchus maculatus* F. (Coleoptera: Chrysomelidae: Bruchinae) on cowpea and *Sitophilus oryzae* L. (Coleoptera: Curculionidae) on wheat were exposed to hypoxia treatments consisting of 1, 3, and 5% oxygen levels for 14 d. Acoustic activity was monitored during the experiment, and insect mortality and grain quality were examined immediately after the hypoxia treatments. Adult emergence was assessed 45 d post-treatment. All three hypoxia treatments eliminated acoustic activity of both species within 4 d. There was neither insect survival for both species nor significant grain damage immediately after 14-d exposure to hypoxia treatments. No adult insects emerged 45 d post-exposure on grains maintained at 1% oxygen level for 14 d. However, at 3 and 5% oxygen levels, there were eggs on cowpea, holes in wheat, and emerging adults for both insect species 45 d post-exposure. Although insect activity ceased within 4 d when hypoxia was maintained below 5%, there is need to explore exposure beyond 14 d for 3 and 5% oxygen levels, to ensure to avoid potential adult emergence from eggs and other insect life stages post-treatments. Maintaining 3–5% hypoxia conditions for a longer duration would ensure insufficient oxygen is available for progeny development.

Modified atmospheres have been studied as potential alternatives to conventional fumigants and protectants against stored-product insect pests since the early 1980s, when low-oxygen treatments gained exemption from tolerance requirements on agricultural commodities in the United States (Fleurat-Lessard 1990, Ofuya and Reichmuth 2002). Applications of modified atmospheres were developed as a result (Villers et al. 2008, Mbata et al. 2009, Murdock et al. 2012, Navarro 2012). Modified atmospheres are created by 1) insect and grain respiration in hermetically sealed environments where free oxygen (O_2_) is depleted, hypoxia or anoxia, and carbon dioxide (CO_2_) is increased, hypercarbia, or 2) flushing with nitrogen (N_2_), CO_2_, or highly oxidative compounds such as ozone. Storage under hypoxic conditions can extend the shelf life of grain by preventing further insect proliferation and deterioration of grain quality (Navarro 2012).

Hermetic storage, an example of modified atmospheres, has been widely used for grain storage in developing countries (Baributsa et al. 2010, Jones et al. 2011, Murdock and Baoua 2014). Various studies have explored the effectiveness of hermetic storage on the control of postharvest pests (Villers et al. 2008, Tefera et al. 2011, Murdock et al. 2012, De Groote et al. 2013, Baoua et al. 2014). As with chemical protectants and fumigants, there is concern that insects will develop tolerance to hypoxia.

Several studies have investigated pest mortality under controlled atmospheres, including the use of high CO_2_ and/or low O_2_ in combination with temperature and relative humidity (RH) modifications (Calderon and Navarro 1980, Donahaye et al. 1996, Mbata et al. 1996, 2000, Ofuya and Reichmuth 1998, Ofuya and Reichmuth 2002). Additional studies have investigated the effect of different CO_2_ and reduced oxygen levels on the mortality of *Sitophilus* spp. and the effect of reduced oxygen levels on *Tribolium castaneum* (Herbst) (Coleoptera: Tenebrionidae) (Soderstrom et al. 1992, Emekci et al. 2002, Carli et al. 2010).

Oxygen levels below 5% have been shown to cause complete mortality of adults and other life stages of insects (Donahaye et al. 1992, Navarro 2012, Njoroge et al. 2018). Further research has sought to explain the specific mechanisms that bring about the death of insects under hypoxia—whether death is due to the depletion of oxygen rather than increase in carbon dioxide (Bailey 1955) or whether death is due to desiccation rather than suffocation (Murdock et al. 2012). While modified atmospheres have been shown to kill stored-product pests within a few days, there is evidence that some insects may survive the effects of reduced oxygen and elevated carbon dioxide (Navarro 2012). Insects compensate for hypoxia by increasing tracheal diameters and the number of tracheoles, by decreasing respiration, and also by reducing their metabolic rates (Herreid 1980; Hochachka 1986; Weyel and Wegener 1996; Jarecki et al. 1999; Henry and Harrison 2004; Zhou et al. 2007, 2008). Before females become immobile, they may lay eggs (Yan et al. 2016), leaving behind progeny that may potentially continue their development and result in infestations if the hypoxic environment is breached.

There are documented cases where oxygen levels increased under hermetic conditions after decreasing below 5%, likely due to unidentified breaches of the hermetic seals (Yan et al. 2017, Kharel et al. 2018). Despite the rise in oxygen, the quality of the stored grain and flour were maintained. This is because insects were dead as oxygen decreased below 5% and the containers remained sealed for at least 90 d. However, in the cases where insects are subjected to short hypoxia treatments and then exposed to normoxia, there is a possibility of pest resurgence if all insect stages are not controlled. Monitoring insects after short hypoxia treatments would be useful to assess whether insects would continue their development.

Acoustic detection has been used previously to assess insect activity through measurements of distinctive trains of sound impulses, namely feeding, mating, and locomotion (Mankin et al. 2008, Kiobia et al. 2015). Monitoring pests subjected to hypoxia using acoustic methods may provide insights into insect behavior under modified atmospheres. Njoroge et al. (2017b, 2018) implemented studies to assess insect acoustic behavior under hermetic conditions. Results showed that acoustic behavior declines progressively with the decrease in oxygen levels. The study revealed that there was a direct relationship between the residual oxygen level and the insects’ acoustic activity. Oxygen levels declined to less than 4% from normoxia within 1 wk, and insect sound burst rates fell below an acoustic detection threshold of 0.02 bursts/s indicating that the insects had ceased feeding. Little is known on the behavior response of insects under controlled hypoxic-induced conditions. Unlike hermetic conditions where oxygen is depleted progressively from normoxia, insect in controlled environments are subjected to immediate hypoxia. Monitoring of insect acoustic behavior under low-oxygen levels will help determine recommendations for insect management under controlled atmospheres.

The objectives of the present study were to assess 1) acoustic behavior of stored-product arthropods [*Callosobruchus maculatus* F. (Coleoptera: Bruchidae) and *Sitophilus oryzae* L. (Coleoptera: Curculionidae)] under low-oxygen (1, 3, and 5%) treatments in controlled atmospheres, 2) insect survival and grain quality immediately after exposure to low-oxygen treatments, and (iii) insect emergence 45 d post-treatments.

## Materials and Methods

### Insect Rearing and Sample Preparation

*Callosobruchus maculatus* and *S. oryzae* used for this experiment were maintained in a Conviron Environmental Chamber (C710, Winnipeg, MB, Canada) at the Department of Entomology, Purdue University. Rearing conditions for both colonies were as follows: temperature, 25 ± 1°C; RH, 40 ± 5%; and photoperiod, 12:12 (L:D) h. *Callosobruchus maculatus* were reared on California black-eyed cowpea variety #8046 (Wax Co., Amory, MS) and *S. oryzae* on soft red winter wheat variety AG1189 (Alumni Seed Co., Romney, IN). Grain for the experiment was disinfested for 14 d at −18°C and then thawed at room temperature 1 d before use. Wide-mouth glass jars were filled with approximately 100 g of fresh cowpea or wheat grain and then infested with ~150 adult *C. maculatus* and *S. oryzae*, respectively. The insects were allowed to lay eggs for 2 h and then removed. The infested grain was incubated, and eggs were allowed to develop until the time of first adult emergence. Newly emerged unsexed adult *C. maculatus* and *S. oryzae* were isolated from their respective colonies using no. 10 sieves. A vacuum aspirator was used to collect insects in batches of 50 for introduction into specific controlled atmosphere treatments.

### Controlled Atmosphere Chamber Assemblage

Airtight controlled atmosphere chambers were made from 9.5-liter aquarium tanks. Three aquarium tanks formed enclosures to hold the insects at oxygen levels of 1, 3, or 5%. These levels were chosen because previous studies have demonstrated only limited lethality of low-oxygen treatments against insects until oxygen levels fall below 5% (Navarro 1978; Njoroge et al. 2017a,b, 2018). The tanks were rectangular cuboid glass containers measuring 30.5 cm × 15.2 cm × 20.3 cm. The aquarium was covered with lightweight shatter-resistant acrylic sheets (synthetic polymer of methyl methacrylate). Four holes were drilled on the acrylic covers using a cordless drill (Black & Decker, Towson, MD) fitted with a 0.75 cm drill bit (Menards Inc, Eau Claire, WI). The holes facilitated the installation of a stainless-steel probe to serve as a waveguide for transmission of vibrational signals from the insects to the acoustic sensor-amplifier system. Rubber stoppers (No. 7) were glued to the acrylic covers after drilling to ensure a firm grip of the waveguide and to help prevent gas leakage. Each tank was covered by an acrylic sheet, sealed using Duct Sealing Compound (Gardner Bender, Milwaukee, WI) and secured firmly with Duct tape (Duck, Avon, OH).

Different low-oxygen environments in the aquarium tanks were obtained by flushing with nitrogen from pressurized cylinders. These mixtures were passed into the aquarium tanks throughout the experiment as needed to ensure the O_2_ levels were maintained at the desired concentrations. Each aquarium tank had two OxyDots (OxySense Inc., Dallas, TX) fitted to facilitate monitoring of O_2_ levels using the OxySense 5250i (OxySense). The gas composition levels to which the test insects were exposed were monitored twice a day to check for fluctuations.

### Experimental Set-up

The experiment involved three oxygen levels (1, 3, and 5% O_2_) and two insect pests (*C. maculatus* and *S. oryzae*). Each treatment was replicated four times. The experimental unit (sample) was a fish tank holding four 120-ml glass containers with perforated lids and each holding either wheat or cowpea infested with 50 individuals of a specific treatment.

### Acoustic Measurement

The acoustic devices were set up as described in Herrick and Mankin (2012). A sensor-preamplifier module (model SP-1L Acoustic Emission Consulting [AEC], Sacramento, CA) was attached at the end of the waveguide (probe) passing through the drilled acrylic covers of the aquarium tanks into the infested grain in the jars. The sensor was connected to an AED-2010 amplifier (AEC, Sacramento, CA). The AED-2010 was connected to a digital audio recorder, Marantz professional [model PMD-561, New York City, NY], which stored the insect signals as wav files on memory cards at a 44.1 kHz sampling rate. Recordings of 1 h each were taken twice a day (morning and evening) for the first 6 d and twice a week for the next 8 d as the activity decreased. Insect sound recordings were performed inside the aquarium tanks in an isolated quiet room at ambient temperature (22–25°C), with fluorescent lighting. The amplification levels were standardized throughout the experiment; consequently, the relative amplitudes are the same for all the oscillograms as described in the following section.

### Acoustic Data Management and Signal Processing

Before further processing, recorded signals were first band-pass filtered between 0.2 and 10 kHz using Raven Lite software (Bioacoustics Research Program 2016). Next, five spectral profiles (mean spectra) were calculated for both *C. maculatus* and *S. oryzae* by DAVIS (Digitize, Analyze, View, Insect Sounds) through fast Fourier transform and other algorithms (Mankin et al. 2011, Herrick et al. 2013) from 10-min prescreened records obtained from noise-free periods. It was necessary to construct different profiles for each species due partly to the larger size of *S. oryzae* and partly to differences in the structure of cowpeas and wheat (Mankin et al. 2018), which produced significant differences in the spectra of the sounds each species produced. DAVIS was further used to conduct automated analyses to distinguish daily insect sounds produced by both *C. maculatus* and *S. oryzae*. Each impulse detected in the daily recordings was assigned to the type from which it had the smallest total mean-square difference (Dosunmu et al. 2014). Impulses whose spectra failed to match any profile within a preset least-squares threshold were discarded. For each sample, the DAVIS program identified and timed groups (trains) of at least three impulses separated by intervals < 200 ms whose spectra matched one of the five insect profile types. These trains, called bursts, have been demonstrated in previous studies to have a high likelihood of having been produced by an insect (Jalinas et al. 2015). The beginning and end time of each train and the number of impulses in the train were stored in a spreadsheet. The rates of bursts, the numbers of impulses per burst, and the rates of impulses in bursts were calculated as in Jalinas et al. (2015).

Responses of both *C. maculatus* and *S. oryzae* to the low-oxygen tensions were assessed in terms of burst rates and the rates of impulses in bursts. Furthermore, statistical analyses were used to compute the differences or similarities among burst rates and rates of impulses in bursts from insects of each species exposed to the different oxygen-level treatments.

### Moisture Content, Grain Quality, and Adult Emergence Assessments

At the beginning and end of the experiment, the moisture content was determined using a handheld grain moisture tester (Dickey-John mini GAC plus moisture tester; DICKEY-john Corporation, Auburn, IL). The numbers of insect damaged wheat and infested cowpea were assessed on 100-grain samples and converted into percentage. Given the low-oxygen conditions, the focus was on qualitative changes of grain (presence of holes and eggs) as opposed to quantitative changes (weight loss). Cowpea seeds were infested with eggs, whereas wheat seeds had holes. The number of surviving adult insects was assessed. After assessing and sieving out the adults exposed to hypoxia for 14 d, cowpea seeds and wheat grain were incubated at 25 ± 1°C and 40 ± 5% RH for 45 d. The total number of adults that had emerged from each container after 45 d was counted and recorded. At the beginning of each of the 1-h acoustic recordings, counts were made of insects visibly in movement near the surface of the 120-ml glass jars containing the treatment samples.

### Statistical Analyses

All data were analyzed using Stata SE Version 12 (StataCorp 2011). Percentage data were arcsine transformed to stabilize variance prior to analysis. Analysis of covariance (ANCOVA) was applied to test the effects of treatment, exposure time, and the interaction of treatment and exposure time. Where the coefficient of the interaction term was significant (*P* ≤ 0.05), analysis of variance (ANOVA) was performed to compare mean rates of bursts, and numbers of surviving insects, damaged grains, and emerging adults among the treatments to assess for day-by-day differences. Means were separated using Bonferroni adjustment at 95% confidence level. In several tables, mean values ± SEM are listed.

## Results

### Acoustic Activity of Adult *C. maculatus* and *S. oryzae* Under Low-Oxygen Environments

**Fig. 1.**
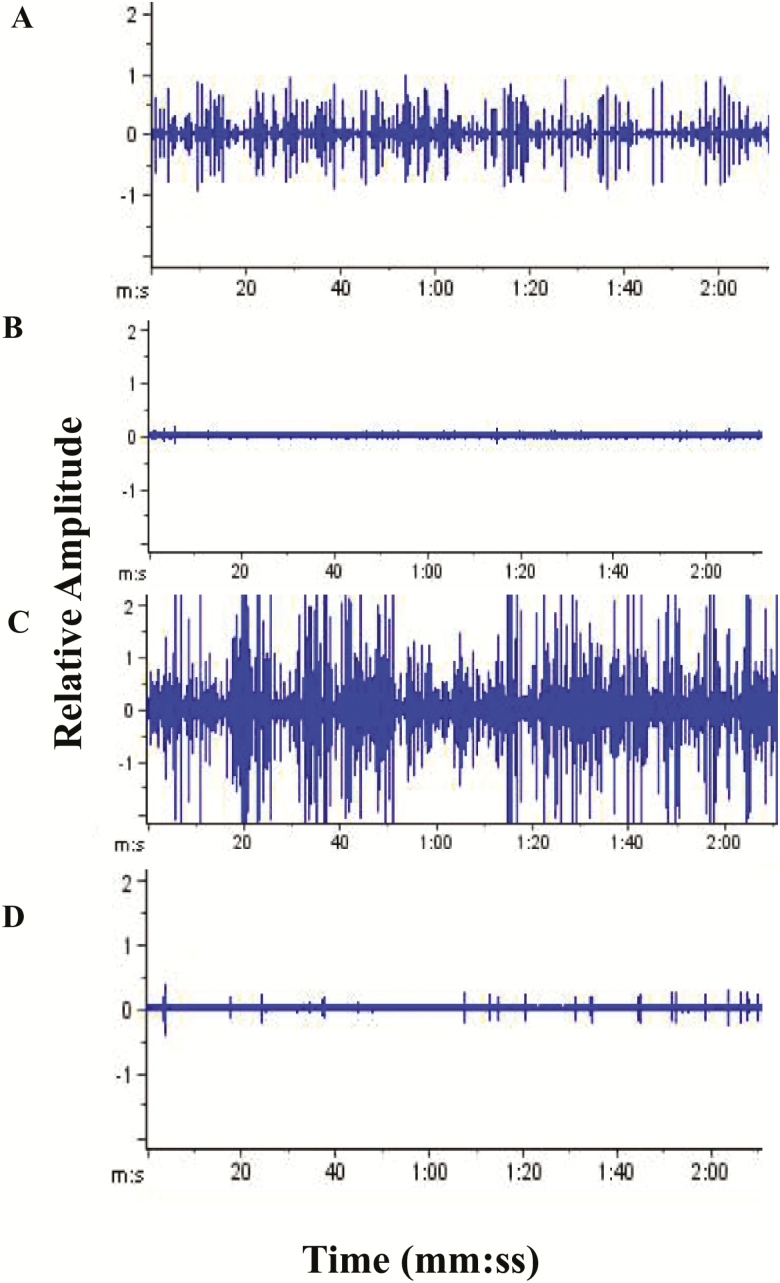
Oscillogram of a 3-min period of impulse patterns recorded from adult infestations of *Callosobruchus maculatus* (A and B) and *Sitophilus oryzae* (C and D) before and after 2-d exposure to controlled atmosphere storage conditions of 1, 3, and 5% oxygen levels.

The acoustic patterns for the two species varied considerably on different days after exposure to low-oxygen environments. The sound impulses showed a broad range of amplitudes, spectral features, and temporal patterns. Figure 1A and C show several impulses in a 2-min recording of adult *C. maculatus* and *S. oryzae*, respectively, under normoxic conditions. The signals grew weaker as time progressed under exposure to the low-oxygen environments during this study. Figure 1B and D nevertheless include several individual impulses and an impulse train in a 2-min recording of adult *C. maculatus* and *S. oryzae*, respectively, after 2-d exposure to 3% oxygen.

**Fig. 2.**
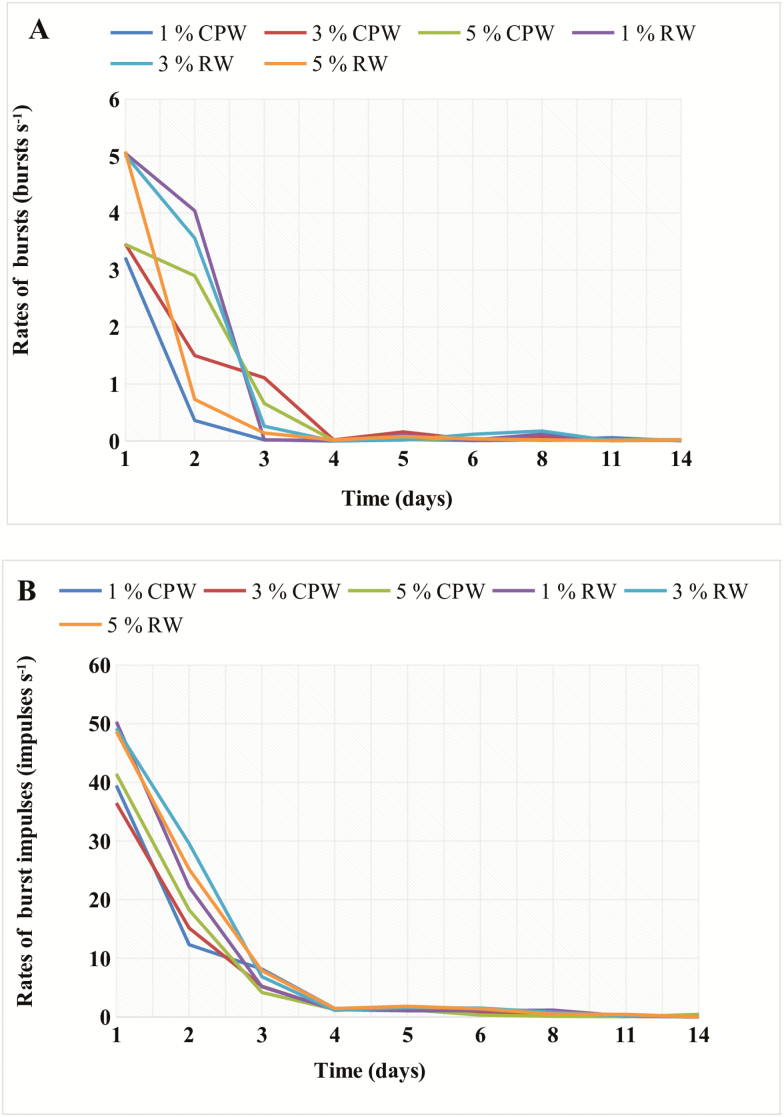
Rates of bursts (A) and rates of burst impulses for (B) *Callosobruchus maculatus* (CPW) and *Sitophilus oryzae* (RW) adults during exposure to controlled atmosphere storage conditions of 1, 3, and 5% oxygen levels for 15 d.

For purposes of analysis, five audibly distinct types of spectra were identified in the prescreening of single, high-quality acoustic recordings of adult *C. maculatus* and *S. oryzae*, respectively. The signal profiles were Broadband, HighF, MidF1, MidF2, and LowF, based on their peak energies and breadths of spectral range. From the 3-min sections identified at the prescreening stage, DAVIS program identified brief 1–10 ms bursts separated by <200 ms and saved them. The rates of bursts and impulses in bursts were computed for each recording. The rates of bursts and rates of impulses in bursts in the different *C. maculatus* and *S. oryzae* treatments declined sharply over the first 4 d of exposure to controlled atmosphere conditions (Fig. 2A and B). ANCOVA analysis showed significant differences in the decline curves of the different treatments and species demonstrated a significant interaction between treatments and exposure time (Table 1). ANOVA analysis showed that there were significant differences (*P* < 0.05) among burst rates for treatments at different points in time on the first 3 d when the insects were most active (Tables 2 and 3). By day 3, there was very little if any acoustic activity recorded for the 1% treatments for both insect species. On day 4, all the insects were ‘acoustically’ dead, i.e., the rates of bursts had fallen below a previously determined threshold for low likelihood of infestation, 0.02 bursts/s (Mankin et al. 2008).

**Table 1.** Analysis of the effects of 1, 3, and 5% oxygen levels, exposure time, and their interaction on the mean rates of bursts, rates of impulses in bursts, and number of impulses per burst insect of *Callosobruchus maculatus* (CPW; *n* = 504) and *Sitophilus oryzae* (RW; *n* = 504) adults during a 14-d exposure period

Parameter	CPW			RW		
	df	*F*	*P*	df	*F*	*P*
Rates of bursts						
Treatment	2	26.26	<0.001	2	12.35	<0.001
Exposure time	8	79.65	<0.001	8	13.24	<0.001
Treatment × Exposure time	16	12.64	<0.001	16	8.99	<0.001
Rates of impulses in bursts						
Treatment	2	22.24	<0.001	2	10.11	<0.001
Exposure time	8	64.52	<0.001	8	18.08	<0.001
Treatment × Exposure time	16	12.76	<0.001	16	3.67	<0.001
Impulses per burst						
Treatment	2	4.26	<0.001	2	4.81	<0.001
Exposure time	8	32.49	0.0021	8	10.28	<0.001
Treatment × Exposure time	16	7.41	<0.001	16	5.08	<0.001

**Table 2.** Analysis of variance of insect sound burst rates produced by *Callosobruchus maculatus* (CPW) adults during the first 4 d of exposure to controlled atmosphere treatment of 1, 3, and 5% oxygen levels (*n* = 96; rates are listed as mean ± SEM)

Treatment	Burst rate (bursts/s)*			
	Day 1	Day 2	Day 3	Day 4
1% O_2_ CPW	3.22 ± 0.31a*	0.36 ± 0.25a	0.02 ± 0.02a	0.0 ± 0.0a
3% O_2_ CPW	3.46 ± 0.97a	1.50 ± 1.10b	1.11 ± 0.24b	0.02 ± 0.01a
5% O_2_ CPW	3.45 ± 0.72a	2.93 ± 0.39c	0.66 ± 0.36ab	0.01 ± 0.01a

*Means in the same column followed by the same letter are not significantly different at *P* ≥ 0.05. Means were separated using Bonferroni adjustment.

**Table 3.** Analysis of variance of insect sound burst rates produced by *Sitophilus oryzae* (RW) adults during the first 4 d of exposure to controlled atmosphere treatment of 1, 3, and 5% oxygen levels (*n* = 96; rates are listed as mean ± SEM)

Treatment	Burst rate (bursts/s)*			
	Day 1	Day 2	Day 3	Day 4
1% O_2_ RW	5.05 ± 0.56a*	4.04 ± 1.16a	0.02 ± 0.01a	0.01 ± 0.01a
3% O_2_ RW	5.02 ± 0.38a	3.56 ± 0.53a	0.26 ± 0.17b	0.0 ± 0.0a
5% O_2_ RW	5.08 ± 0.74a	0.73 ± 0.06b	0.14 ± 0.03b	0.02 ± 0.01a

*Means in the same column followed by the same letter are not significantly different at *P* ≥ 0.05. Means were separated using Bonferroni adjustment.

The cessation of acoustic activity depended on the oxygen level and exposure time as shown in Fig. 2A, for rates of bursts, and in Fig. 2B, for rates of impulses in bursts (burst impulses). Insect activity monitoring was stopped at 14 d. The burst rates in all treatments decreased steadily during the first 3–4 d after which few bursts were recorded. The decline was fastest in the 1 and 3% treatments for both insect species. After day 5, no insect activity was observed and the insects were presumed dead. The time taken until cessation of acoustic activity ranged from 2.9–3.2 d for *C. maculatus* and 3.2–4.1 d for *S. oryzae* under 1, 3, and 5% low-oxygen treatments (Table 4).

**Table 4.** Time after onset until cessation of acoustic activity *of Callosobruchus maculatus* (CPW) and *Sitophilus oryzae* (RW) under 1, 3, and 5% oxygen-level treatments

Treatment	Mean time (d) ± SEM to cease acoustic activity*	
	CPW	RW
1% O_2_	2.9 ± 0.37*	3.2 ± 0.21
3% O_2_	3.5 ± 0.53	4.0 ± 0.14
5% O_2_	3.8 ± 0.24	4.1 ± 0.45

*Means within the same insect species were not significantly different among the oxygen levels.

### Insect Survival and Grain Quality Immediately After Low-Oxygen Treatments

After 14 d, the tanks were opened to assess moisture content, surviving adults, wheat grains with holes, and eggs on cowpeas. Grain moisture levels were 12% for cowpea and 8% for wheat. They remained the same by the end of the experiment (data not shown). Insect activities observable through the glass declined from normal to occasional weak movements, decreasing day by day and ceasing by the fourth day of treatment. At the end of 14 d, there were no surviving adults in any treatments. Immediately after 14 d, there was no significant difference (*P* > 0.05) among hypoxia treatments on grain quality of wheat or cowpea (Table 5). Infestation of cowpea grain with eggs ranged from 3.49 to 3.78% whereas for wheat, it ranged from 7.56 to 8.08%.

**Table 5.** Analysis of variance of percent cowpea grains infested with *Callosobruchus maculatus* (CPW) and wheat grains damaged by *Sitophilus oryzae* (RW) after 14-d exposure to 1, 3, and 5% oxygen levels and insect emergence after 45-d incubation (*n* = 16; rates are listed as mean ± SEM)

Treatment	% infested cowpea grains	% damaged wheat grains	Number of emerging adults 45 d post-treatment*	
			CPW	RW
1% O_2_	3.49 ± 0.39a*	8.08 ± 0.43a	0.0 ± 0.0a	0.0 ± 0.0a
3% O_2_	3.78 ± 0.23a	7.68 ± 0.40a	10.67 ± 2.12b	9.67 ± 4.47b
5% O_2_	3.43 ± 0.25a	7.56 ± 0.29a	14.44 ± 3.13c	16.56 ± 5.07c

*Means within the same column followed by the same letter are not significantly different at *P* ≥ 0.05. Means were separated using Bonferroni adjustment.

### Insect Development 45 d Post-treatment

After 45 d post-treatment incubation (Table 5), however, there were significant differences in adult emergence among hypoxia treatments for both *C. maculatus* and *S. oryzae* insect species (*P* < 0.05). For both species, treatments exposed to 1% oxygen had no emerging insects after 45 d post-treatment incubation despite having visible eggs or damage on the cowpea and wheat, respectively. Similar numbers of adults emerged from the 3 and 5% treatments for both *C. maculatus* and *S. oryzae* treatments.

## Discussion

### Acoustic Activity of Adult *C. maculatus* and *S. oryzae* Under Low-Oxygen Environments

Acoustic methods provide a means for monitoring insect behavior and have been previously used to assess infestations of both adult and pre-emergent insect stages (Schwab et al. 2005, Mankin et al. 2011, Eliopoulos et al. 2015, Kiobia et al. 2015). Reductions in insect activity typically are correlated with reductions in rates of movement and feeding sounds; consequently, the acoustic activity can be monitored to assess the behavioral response of insects to low-oxygen treatments (Jalinas et al. 2015, 2017; Njoroge et al. 2017a,b, 2018).

In this study, we assessed how 1, 3, and 5% oxygen levels affected infestations of adult *C. maculatus* and *S. oryzae* on stored cowpea and wheat grains, respectively, over a 14-d period. The temporal and spectral patterns of their signals were comparable with those observed previously for stored-product insects subjected to hermetic conditions (Njoroge et al. 2017a,b, 2018), with the acoustic activity of both *C. maculatus* and *S. oryzae* decreasing over time after exposure to the controlled atmosphere conditions. Reductions in acoustic activity were measured as decreases in both the sound burst rates and the rates of burst impulses (Fig. 2). This decline culminated in total cessation of acoustic activity within 3–4 d of exposure to low-oxygen levels for both species leading to insects becoming immobile as we could see through the glass jars. The duration until cessation of activity was similar for the three low-oxygen treatments in this study. Njoroge et al. (2017b, 2018) showed that when oxygen levels in hermetic environments reach below 5%, insect acoustic activities cease. These studies determined that the decline of burst rates to below 0.02 bursts/s, associated with 5% or less oxygen levels, is a threshold below which a low likelihood of infestation is predicted acoustically. Based on these research findings, it is clear that 5% oxygen level or less will stop insect activities during grain storage under controlled environments. These findings corroborate laboratory and field results of several experiments. Oxygen depletion below 5% has shown to be effective in eliminating insect infestations during grain storage in hermetic bags (Baoua et al. 2014, Njoroge et al. 2014). Similarly, low-oxygen levels below 5% provided a lethal environment for various postharvest pests such as *Ephestia cautella* (Walker) (Lepidoptera: Phycitidae) and *T. castaneum* (Herbst) (Navarro 1978). Under these conditions, hermetic storage can produce the same effects as controlled atmospheres on stored-product insects.

The time required for insect acoustic activities to cease varies under modified atmosphere systems as it depends on several factors such as total oxygen available and/or insect populations. Though it took only 3–4 d to reach quiescence in this study, the time required to kill adult insects under controlled environment in this experiment was mostly dependent on oxygen concentration and less on insect species. Hypoxia treatments eliminated acoustic activity of both insect species over a similar time range probably because they have similar oxygen-dependent physiological processes. Kharel et al. (2019) showed that the time required to kill adult *T. castaneum* (Coleoptera: Tenebrionidae) under controlled environment varied with hypoxia treatments: 2 and 4% oxygen levels achieved complete and 90% mortality, respectively, in 15 d. Under hermetic storage conditions, however, the time required to kill insects depends on how fast oxygen is depleted. Oxygen depletion is determined by several factors including level of infestation, initial quantity of oxygen available, and environmental conditions such as temperature and RH. Njoroge et al. (2018) conducted an experiment to assess the effect of cowpea bruchid population (25, 50, and 100 adults) and available oxygen (0.5- and 1.0-liter jars) on oxygen consumption and acoustic activities. The study revealed that cessation of insect activities happened when oxygen level reached 4% after 3–11 d exposure to hermetic storage conditions. Less time is required to reach the cessation of insect acoustic activity under controlled environments than in hermetic storage conditions.

### Insect Survival and Grain Quality Immediately After Low-Oxygen Treatments

Assessment immediately after exposure to hypoxia treatments for 14 d revealed no adult *S. oryzae* survived. These findings are in agreement with results of Kharel et al. (2019) who showed that exposure of *T. castaneum* to 2% or less for 15 d resulted in no adult survival. However, exposure to higher oxygen levels such as 4 and 8% did not result in complete mortality. We did not expect to find any live *C. maculatus* as they have a short life cycle of 10–12 d (Yan et al. 2016). Assessment of the grain immediately after low-oxygen exposure showed the presence of grain damage on wheat for all the treatments. This appears to be the result of damage by *S. oryzae* adult insects before the cessation of activities (within 3–4 d of exposure). For cowpea, there were eggs laid on the grains and no other damage was observed. We used qualitative assessment such as grain with eggs and holes because previous studies have shown minimal weight losses when grain is stored under hermetic conditions (Njoroge et al. 2017b). No change in weight loss was expected in this study, and hence we assessed qualitative measures that are indicative of potential future progeny development when grain is returned to normoxic conditions. Previous studies have shown that insect activity and grain infestation and damage are reduced at oxygen levels below 5% (Emekci et al. 2002; Margam 2009; Yan et al. 2016; Njoroge et al. 2017b, 2018).

### Insect Development 45 d Post-treatment

A few adults were present in grain incubated for 45 d after exposure to 3 and 5% oxygen levels for 14 d. Consequently, when assessing the ultimate efficacy of controlled environments, initial mortality is not the only parameter of importance especially if the airtight conditions are not maintained after initial exposure to hypoxia. The presence or absence of eggs and surviving adults post-treatment need to be assessed to ensure that there is no pest resurgence after the airtight system is compromised.

Some studies have demonstrated that low oxygen (2–5%) can suppress egg-laying behavior and adult emergence of *C. maculatus* (Yan et al. 2016). In our study, there were no surviving adults immediately after 14 d of hypoxia treatments; the eggs laid were few, and some eggs did not hatch 45 d post-treatment. These findings were consistent with Cheng et al. (2012) who found that decreased O_2_ and elevated CO_2_ affected survival, development, and gene expression in cowpea bruchids. They found that below 2% oxygen, bruchids slow down or stop ovipositing. Furthermore, our results showed no adult emergence from 1% oxygen level after 45 d post-treatment. This result corroborates previous findings that show exposure of *C. maculatus* and *T. castaneum* eggs to oxygen levels below 2% causes up to 100% egg mortality (Cheng et al. 2012, Kharel et al. 2019). Though 3 and 5% resulted in a few live adults 45 d post-treatment, they might have affected the development of the insects. Kharel et al. (2019) showed that adult developmental time was increased by 18 and 15 d when was young larvae of *T. castaneum* were exposed 4 and 8% oxygen levels, respectively. Carli et al. (2010) showed complete inhibition of progeny development when *Sitophilus* spp. were exposed to CO_2_ atmospheres for 30 d. Extending exposure time beyond 14 d at 3 and 5% oxygen levels may result in complete mortality post-treatment.

In conclusion, exposure of *C. maculatus* and *S. oryzae* to hypoxia of 5% or below halts insect activity within 4 d. Controlled atmosphere of 1% oxygen level for 14 d is effective for achieving complete adult mortality of *C. maculatus* and *S. oryzae* and preventing insect resurgence post-treatment. Maintaining oxygen levels at 3 and 5% will contribute to the death of adult insects within 14 d but may lead to insect development post-treatment if airtight conditions are breached. Consequently, maintaining low-oxygen conditions for a longer duration would ensure insufficient oxygen is available for progeny development and thus protect the stored grain from further deterioration.
